# Automatic segmentation of the thalamus using a massively trained 3D convolutional neural network: higher sensitivity for the detection of reduced thalamus volume by improved inter-scanner stability

**DOI:** 10.1007/s00330-022-09170-y

**Published:** 2022-10-20

**Authors:** Roland Opfer, Julia Krüger, Lothar Spies, Ann-Christin Ostwaldt, Hagen H. Kitzler, Sven Schippling, Ralph Buchert

**Affiliations:** 1 jung diagnostics GmbH, Hamburg, Germany; 2 Institute of Diagnostic and Interventional Neuroradiology, University Hospital Carl Gustav Carus, Technische Universität Dresden, Dresden, Germany; 3 Multimodal Imaging in Neuroimmunological Diseases (MINDS), Center for Neuroscience Zurich (ZNZ), Federal Institute of Technology (ETH), University of Zurich, Zürich, Switzerland; 4 Department of Diagnostic and Interventional Radiology and Nuclear Medicine, University Medical Center Hamburg-Eppendorf, Martinistr. 52, 20246 Hamburg, Germany

**Keywords:** Thalamus, Magnetic resonance imaging, Neural networks, Multiple sclerosis

## Abstract

**Objectives:**

To develop an automatic method for accurate and robust thalamus segmentation in T1w-MRI for widespread clinical use without the need for strict harmonization of acquisition protocols and/or scanner-specific normal databases.

**Methods:**

A three-dimensional convolutional neural network (3D-CNN) was trained on 1975 T1w volumes from 170 MRI scanners using thalamus masks generated with FSL-FIRST as ground truth. Accuracy was evaluated with 18 manually labeled expert masks. Intra- and inter-scanner test-retest stability were assessed with 477 T1w volumes of a single healthy subject scanned on 123 MRI scanners. The sensitivity of 3D-CNN-based volume estimates for the detection of thalamus atrophy was tested with 127 multiple sclerosis (MS) patients and a normal database comprising 4872 T1w volumes from 160 scanners. The 3D-CNN was compared with a publicly available 2D-CNN (FastSurfer) and FSL.

**Results:**

The Dice similarity coefficient of the automatic thalamus segmentation with manual expert delineation was similar for all tested methods (3D-CNN and FastSurfer 0.86 ± 0.02, FSL 0.87 ± 0.02). The standard deviation of the single healthy subject’s thalamus volume estimates was lowest with 3D-CNN for repeat scans on the same MRI scanner (0.08 mL, FastSurfer 0.09 mL, FSL 0.15 mL) and for repeat scans on different scanners (0.28 mL, FastSurfer 0.62 mL, FSL 0.63 mL). The proportion of MS patients with significantly reduced thalamus volume was highest for 3D-CNN (24%, FastSurfer 16%, FSL 11%).

**Conclusion:**

The novel 3D-CNN allows accurate thalamus segmentation, similar to state-of-the-art methods, with considerably improved robustness with respect to scanner-related variability of image characteristics. This might result in higher sensitivity for the detection of disease-related thalamus atrophy.

**Key Points:**

*• A three-dimensional convolutional neural network was trained for automatic segmentation of the thalamus with a heterogeneous sample of T1w-MRI from 1975 patients scanned on 170 different scanners.*

*• The network provided high accuracy for thalamus segmentation with manual segmentation by experts as ground truth.*

*• Inter-scanner variability of thalamus volume estimates across different MRI scanners was reduced by more than 50%, resulting in increased sensitivity for the detection of thalamus atrophy.*

**Supplementary Information:**

The online version contains supplementary material available at 10.1007/s00330-022-09170-y.

## Introduction

There is growing interest in MRI-based volumetry of the thalamus, for example, in the management of patients with multiple sclerosis (MS). The thalamus is among the brain structures with the earliest signs of atrophy detectable in MRI in the course of MS [1], and MRI-based thalamus volume is a promising marker to predict the transition from clinically isolated syndrome to clinically definite MS [2]. Furthermore, thalamus atrophy is among the strongest predictors of cognitive impairment in MS [3] and therefore can serve as a surrogate outcome for cognition in MS therapy trials [4, 5].

Manual delineation by an expert is the ground truth for MRI-based volumetry of the thalamus. Methods for automatic thalamus segmentation have been developed to facilitate thalamus volumetry [6–9], as manual segmentation by an expert is not compatible with the busy clinical routine at most sites. However, automatic brain volumetry methods are sensitive to the MRI scanner platform and details of the acquisition protocol [10–14]. This limits the sharing of normal databases and/or cutoffs between sites and/or scanners, which in turn detracts from the utility of automatic brain volumetry for widespread clinical use. Thus, there is a great need for automatic brain volumetry methods that are more robust with respect to scanner-related variability of image characteristics in MRI (e.g., gray-to-white matter contrast, signal-to-noise).

Convolutional neural networks (CNNs) outperform conventional machine learning approaches in many medical imaging tasks including MRI-based brain volumetry [15, 16]. CNNs are also particularly promising in non-harmonized multi-site settings, since they can be made robust with respect to scanner-related variability by training with a heterogeneous dataset covering the whole range of image characteristics encountered in the considered multi-site setting [17].

Against this background, the present study trained a 3D-CNN for automatic thalamus segmentation in T1w-MRI on a large heterogeneous dataset. The 3D-CNN was compared with a publicly available 2D-CNN (FastSurfer [16]) and FSL [18] with respect to accuracy, intra- and inter-scanner test-retest stability, and sensitivity to detect thalamus atrophy.

## Materials and methods

All datasets used in this study comprise 3D gradient echo T1w volume images of the brain acquired with scanner-specific acquisition protocols recommended by the manufacturer for MRI-based brain volumetry. Slice thickness ranged between 0.9 and 1.3 mm. TE time ranged between 1.7 and 5.1 ms (mean 3.1 ± 0.6 ms). TR time ranged between 4.7 and 25 ms (mean 10.1 ± 6.0 ms) for GE and Philips scanners, and between 4.5 and 3000 ms (mean 1837 ± 459 ms) for Siemens scanners.

A summary of the datasets is provided in Table 1.

**Table 1:** Overview of the datasets

	No. of scans	No. of patients	No. of scanners	No. of Siemens scanners	No. of Philips scanners	No. of GE scanners	No. of 3T scanners	No. of 1.5T scanners	Age [years] mean (std) range
Training dataset	1975	1975	170	110	41	19	124	46	51.8 (19.5) [20, 90]
IBSR	18	18	2	1	0	1	1	1	38.3 (22.4) [7, 71]
FTHP	477	1	123	75	36	12	35	88	49.5 (0.4) [49, 51]
MS patients	127	127	2	1	1	0	1	1	38.4 (9.7) [20, 63]
Normal database	4872	4872	160	109	37	14	44	116	49.7 (16.8) [20, 90]

*IBSR*, Internet Brain Segmentation Repository; *FTHP*, frequently traveling human phantom; *MS*, multiples sclerosis

### Dataset for the training of the 3D-CNN

The training dataset comprised 1975 clinical T1w volume images of 1975 different patients from 170 different MRI scanners. The volume images were randomly selected from a larger dataset to achieve a rather uniform distribution of patient age between 20 and 90 years. No additional eligibility criteria were applied to guarantee that the training set covered the whole range of T1w volume images encountered for MRI-based volumetry in clinical routine. In particular, there were no eligibility criteria with respect to the patients’ health status.

### IBSR dataset for the assessment of segmentation accuracy

Eighteen T1w volume images together with masks of the subcortical regions including the thalamus manually delineated by experts freely available from the Internet Brain Segmentation Repository (IBSR, https://www.nitrc.org/projects/ibsr/) were used for the assessment of segmentation accuracy.

### Frequently traveling human phantom (FTHP) dataset for the assessment of intra- and inter-scanner variability

T1w volume images of a single healthy middle-aged male subject who completed 123 imaging sessions on 123 different MRI scanners were used for the assessment of intra- and inter-scanner variability. Most imaging sessions comprised three to five repeat acquisitions (without repositioning) resulting in a total of 477 volume images of the same healthy subject within a period of about 2.5 years.

The FTHP dataset is freely available for research purposes (https://www.kaggle.com/datasets/ukeppendorf/frequently-traveling-human-phantom-fthp-dataset).

### MS patient dataset to test sensitivity of single subject analysis

T1w volume images of 127 MS patients were included retrospectively to assess the sensitivity of the 3D-CNN to detect thalamus atrophy. Thirty-three of them had been enrolled in a clinical study at the Institute of Diagnostic and Interventional Neuroradiology of the University Hospital Carl Gustav Carus, Dresden, Germany (age 42.2 ± 10.1 years, Expanded Disability Status Scale 2.7 ± 1.6, disease duration 5.2 ± 4.8 years). The remaining 94 MS patients had participated in an observational study at the University Hospital of Zurich, Switzerland (age 37.3 ± 8.9 years, Expanded Disability Status Scale 1.3 ± 1.3, disease duration 2.7 ± 4.5 years).

### Normal database for single subject analysis

A sample of 4872 T1w-MRI from 4872 different patients acquired on 160 different MRI scanners for unspecific symptoms (headache, dizziness) was used as normal database for single subject analyses. None of the patients had a history of or currently ongoing neurological or psychiatric condition. All volume images were free of abnormalities beyond those expected for the patients’ age based on visual inspection by the local radiologist.

### Ethics approval and consent to participate

The MRI data of the training set and of the normal database had been transferred to jung diagnostics GmbH under the terms and conditions of the European General Data Protection Regulation for remote image analysis. Subsequently, the data had been anonymized. The need for written informed consent for the retrospective use of the anonymized data in the present study was waived by the ethics review board of the general medical council of the state of Hamburg, Germany.

Ethics approval for the retrospective use of the FTHP dataset was obtained from the same ethics review board. The single subject had given written informed consent.

The MS patient dataset comprised data from two prospective studies that had been approved by the local ethics committees. All patients had given written informed consent. This included the retrospective use of the data for the present study.

### Thalamus segmentation with FSL

The FIRST module of the FSL Software (FSL; version 6.0.2; http://fsl.fmrib.ox.ac.uk/fsl) provides binary masks of deep gray matter structures including the thalamus [18].

### 3D-CNN for thalamus segmentation

**Fig. 1:**
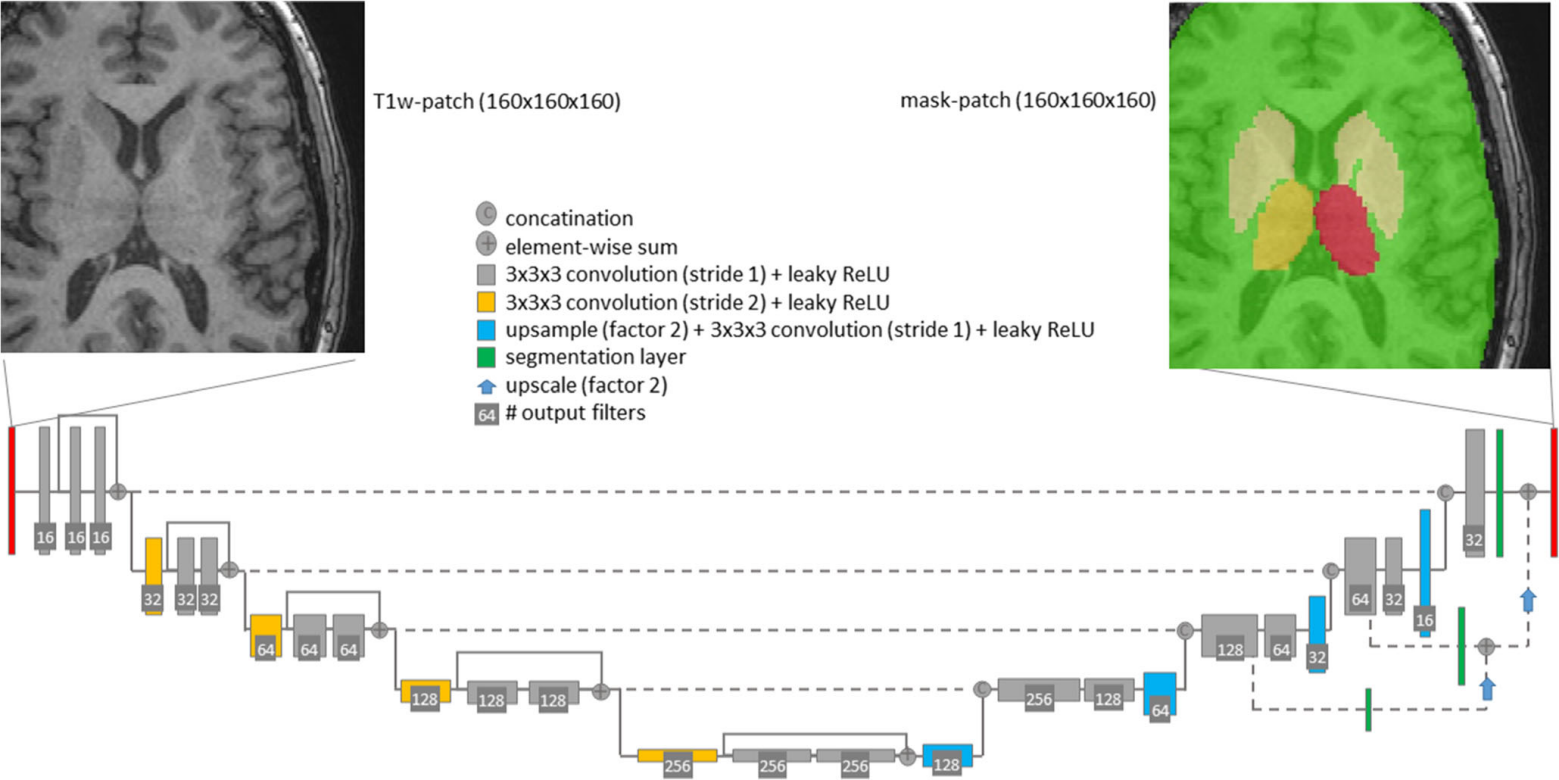
The proposed 3D-CNN U-net: A patch-wise approach with a patch size of 160 × 160 × 160 cubic voxels of 1-mm edge length was used. A fully convolutional encoder-decoder architecture with 3D convolutions, residual-block-connections, and four reductions of the feature map size was employed. The network was trained for simultaneous segmentation of the left and right thalamus, remaining deep gray matter structures, and remaining total intracranial volume

A 3D-CNN-U-net architecture [19, 20] recently introduced for the segmentation of white matter hyperintensities in T2w-MRI [15, 21] was used (Fig. 1). The network was trained for the simultaneous segmentation of the right thalamus, left thalamus, remaining deep gray matter structures, and remaining total intracranial volume (TIV) (Fig. 1). The additional classes (beyond the left and right thalamus) were introduced to provide regional context which has been proven beneficial for other segmentation tasks [22].

The ground truth for the deep gray matter structures was obtained with FSL. The ground truth for the TIV was derived using a validated algorithm [23] based on the Statistical Parametric Mapping framework (version SPM12, https://www.fil.ion.ucl.ac.uk/spm/software/spm12/) [24]. The 3D-CNN was trained using massive data augmentation including simulation of random bias fields and statistical noise in addition to standard augmentation techniques (rotation, flipping). Manual quality control of ground truth segmentation was not performed.

A more detailed description of the 3D-CNN architecture, the training, and the data augmentation is given in the Supplementary Material.

The trained 3D-CNN is available from the authors upon request under a non-disclosure agreement for non-commercial use.

### Thalamus segmentation with FastSurfer

The recently introduced FastSurfer pipeline [16] deploys a 2D-U-net-CNN trained on masks automatically generated with FreeSurfer [25]. FastSurfer is freely available (https://github.com/Deep-MI/FastSurfer). FastSurfer was used with default parameter settings and the pre-trained networks.

### Segmentation accuracy

The accuracy of bilateral thalamus segmentation by 3D-CNN, FastSurfer, and FSL in the IBSR dataset was characterized by the Dice similarity coefficient and the Hausdorff distance relative to the manual expert delineation. Analysis of variance for repeated measurements was used to compare the Dice coefficient and the Hausdorff distance between 3D-CNN, FastSurfer, and FSL. Bland-Altman plots were used to compare bilateral thalamus volume (THALV) estimates between the automatic methods and the manual ground truth.

### Intra- and inter-scanner variability

Intra- and inter-scanner variability of THALV were assessed in the FTHP dataset. To measure intra-scanner variability, the residuals with respect to the mean THALV of all repeat scans with the same scanner (without repositioning) were computed for each THALV estimate. The intra-scanner residuals were pooled into a single vector (of length 477). To measure inter-scanner variability, first the mean values of THALV were computed for each scanner and pooled into a single vector (of length 123). Then, the mean overall mean values were subtracted element-wise from this vector, resulting in a vector of inter-scanner residuals with zero mean. Levene’s test for equality of variances was used for pairwise comparison of the variance of intra- and inter-scanner residuals between 3D-CNN, FastSurfer, and FSL. Bonferroni correction was used to adjust the significance level for the number of pairwise tests; that is, *p* values smaller than 0.05/3 = 0.017 were considered statistically significant. The 50th and 95th percentiles of the absolute value of intra- and inter-scanner residuals were determined to further characterize intra- and inter-scanner variability.

### Single subject analysis

Regional brain volumes including THALV are strongly correlated with head size [26] and subject age [27]. The TIV estimated in T1w-MRI using a 3D-CNN specifically trained for this purpose was used as surrogate of head size. Removing between-subjects variability associated with TIV and age can improve the power of MRI-based brain volumetry to detect disease-related alterations. In the present study, the following procedure was used to account for the impact of TIV and age on THALV. First, the THALV-TIV-age relationship was modeled with THALV = *a* ∗ TIV^2^ + *b* ∗ ag*e*^2^ + *c* ∗ TIV ∗ age + *d* ∗ TIV + *e* ∗ age + *f* by minimizing the sum of squared differences in the normal database. Then, the residual of THALV with respect to this model, denoted resTHALV, was computed for each subject in the normal database, that is, resTHALV = THALV − (*a* ∗ TIV^2^ + *b* ∗ age^2^ + *c* ∗ TIV ∗ age + *d* ∗ TIV + *e* ∗ age + *f*﻿) [28]. Since regression can be affected by outliers, a two-step approach was used. After the first regression, outliers were identified and removed from the second and final regression. Subjects with resTHALV < lower quartile − 1.5*inter-quartile range of resTHALV in the normal database or resTHALV > upper quartile + 1.5*inter-quartile range were considered outliers.

The 95% confidence interval (95% CI) of resTHALV was computed as [−1.96*std, +1.96*std], where std is the standard deviation of resTHALV relative to the final THALV-TIV-age model in the normal database excluding outliers.

Modeling of the THALV-TIV-age relationship in the normal database was performed separately for THALV estimates from 3D-CNN, FastSurfer, and FSL. The same TIV estimate was used for the three thalamus segmentation methods.

For single subject analysis of THALV in MS patients, a two-sample test approach was used, which is more conservative than a one-sample test approach [29]. More precisely, the residual resTHALV of the patient’s THALV was computed with respect to the THALV-TIV-age model (obtained in the normal database) using the age and the individual TIV estimate for the patient. The 95% CI of the patient’s resTHALV was approximated by the 95% CI of the inter-scanner variability in the FTHP. The patient’s THALV was considered reduced if resTHALV including its 95% CI was below the 95% CI of resTHALV in the normal database (Supplementary Fig. 1) [30]. The proportion of MS patients with reduced THALV was compared between 3D-CNN, FastSurfer, and FSL.

## Results

Each of the two steps for the 3D-CNN training took approximately 2 days using a 2.4GHz CPU (Intel Xeon Silver 10-Core) with a GPU Quadro P5000 with 16GB memory. Computation time for thalamus segmentation was approximately 1 min with 3D-CNN and FastSurfer (4GB GPU memory needed), and approximately 5 min with FSL.

Visual inspection did not reveal clear failures of thalamus segmentation in any of the test cases (IBSR, FTHP, and MS dataset) with any of the automatic methods.

### Segmentation accuracy

**Fig. 2:**
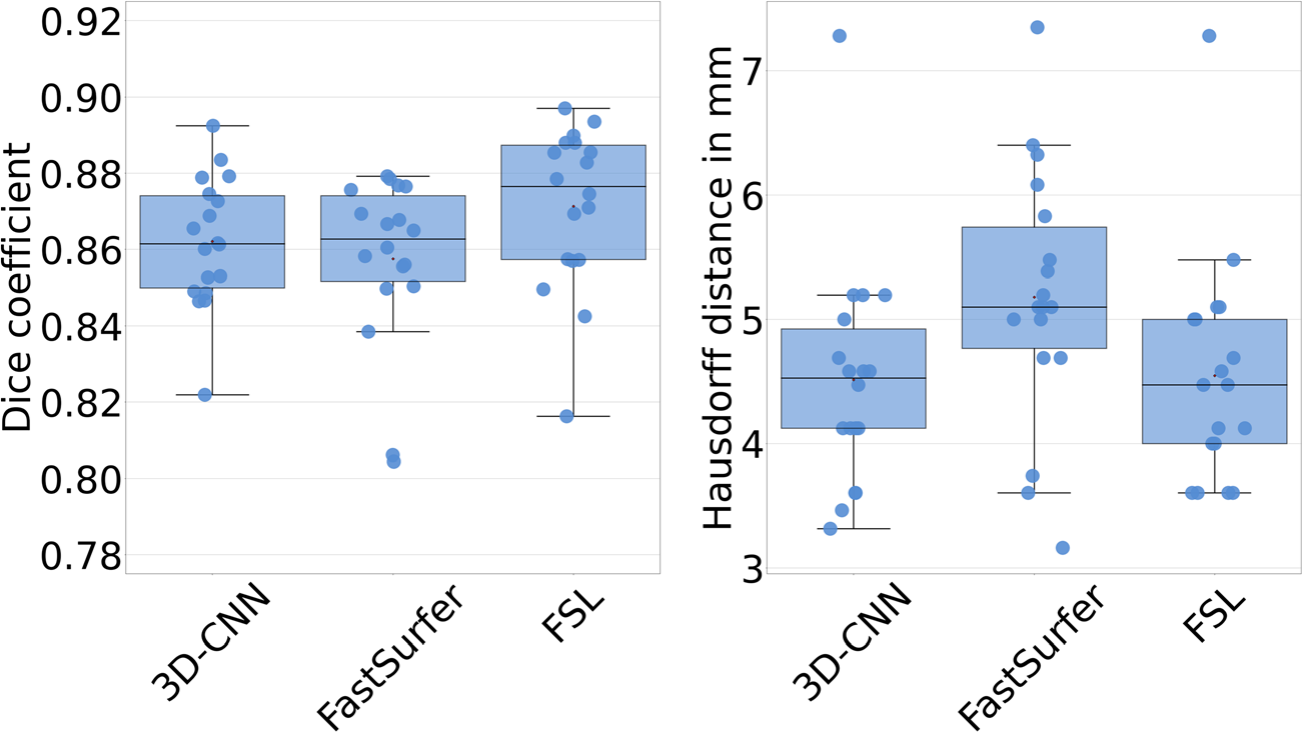
Dice similarity coefficient (left) and Hausdorff distance (right) of thalamus segmentation with 3D-CNN, FastSurfer, and FSL relative to the manually derived expert masks in the IBSR dataset

**Fig. 3:**
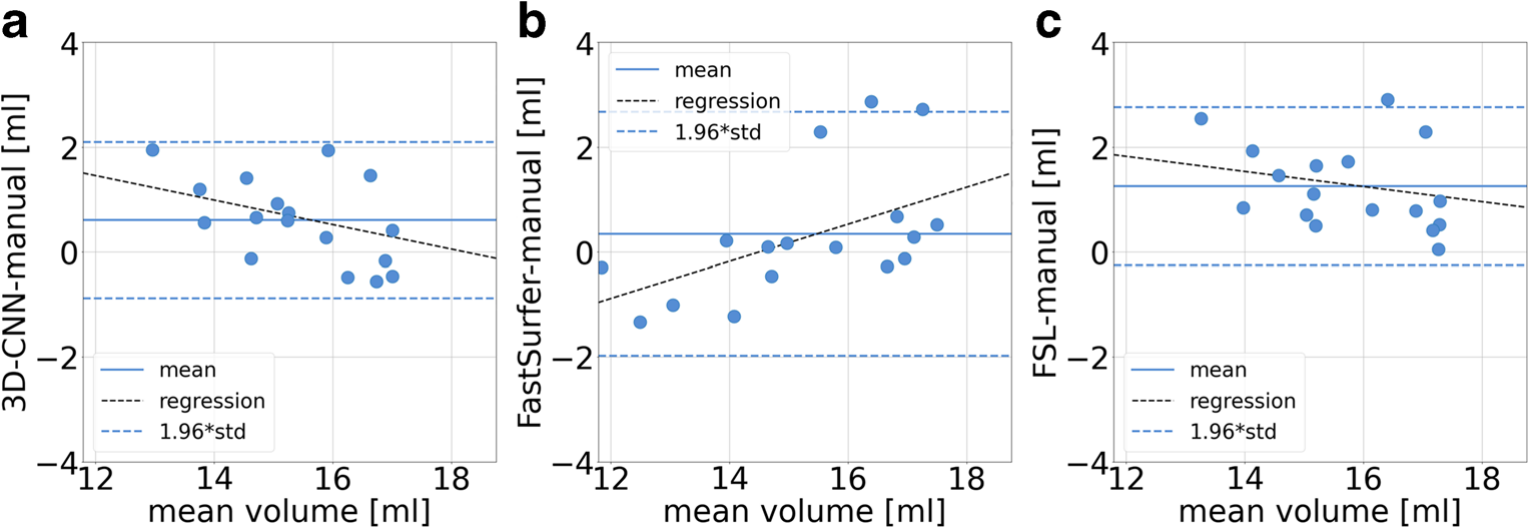
Bland-Altman plots of automatic thalamus volume estimates from 3D-CNN (**a**), FastSurfer (**b**), and FSL (**c**) in comparison with the manually derived expert masks in the IBSR dataset

**Fig. 4:**
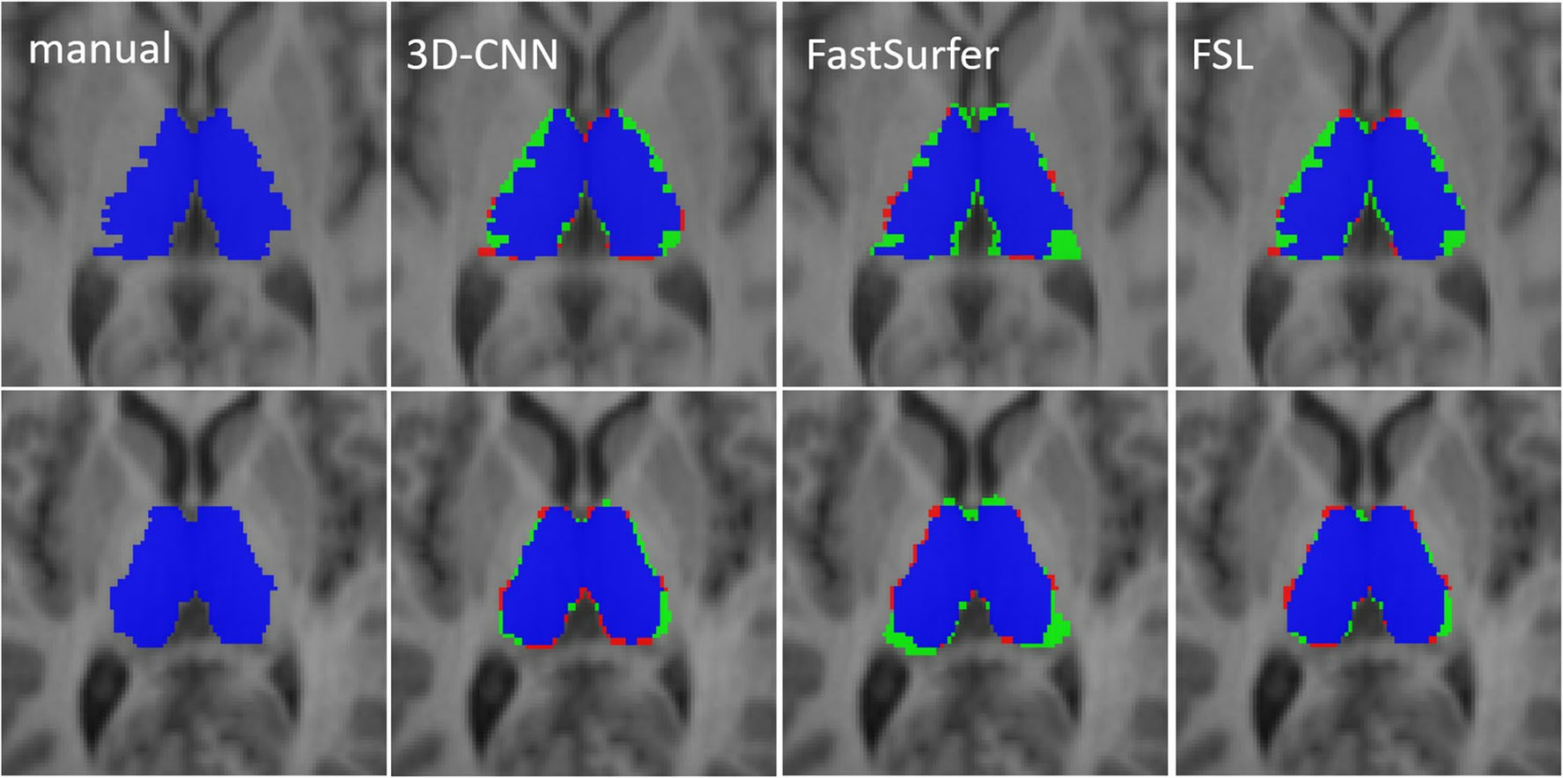
Manually delineated ground truth thalamus mask (left column) and the masks automatically generated with each of the three software tools for two cases from the ISBR dataset (first row: case 6, second row: case 18). Case 6 had the highest Hausdorff distance between the ground truth and the 3D-CNN (7.28mm), and case 18 the lowest (3.46 mm). True positive voxels of the automatic masks are shown in blue, false positive voxels in green, and false negative voxels in red. The examples illustrate the observed overestimation of the thalamus volume relative to the manual ground truth by all automatic methods. The examples also indicate that the automatic methods tend to generate smoother segmentation surfaces than manual segmentation

The results on segmentation accuracy are summarized in Table 2 and Figs. 2 and 3. The Dice coefficients of the automatically generated thalamus masks relative to the manual expert delineation did not differ between 3D-CNN and FastSurfer; they were slightly higher for FSL (3D-CNN and FastSurfer 0.86 ± 0.02, FSL 0.87 ± 0.02; ANOVA with Greenhouse-Geisser non-sphericity correction *p* = 0.003; 3D-CNN or FastSurfer versus FSL *p* = 0.002, Cohen’s *d* = 0.50; 3D-CNN versus FastSurfer *p* = 0.274, *d* = 0.01; Fig. 2). The Hausdorff distance of the automatically generated thalamus masks relative to the manual ground truth masks did not differ between 3D-CNN and FSL; it was slightly higher for FastSurfer (4.51 ± 0.91 mm, 4.55 ± 0.90 mm, and 5.18 ± 1.03 mm; ANOVA with Greenhouse-Geisser non-sphericity correction *p* = 0.033; 3D-CNN versus FastSurfer *p* = 0.023, *d* = 0.68; 3D-CNN versus FSL *p* = 0.848, *d* = 0.04; FastSurfer versus FSL *p* = 0.054, *d* = 0.65; Fig. 2). All automatic methods overestimated THALV on average compared to the expert delineation (*p* < 0.0005; Fig. 3). This was mainly driven by overestimation of small THALV for 3D-CNN and FSL, and by overestimation of large THALV for FastSurfer (Fig. 3). Examples are shown in Fig. 4.

**Table 2:** Accuracy of automatic thalamus segmentation with 3D-CNN, FastSurfer, and FSL compared to the manual expert delineation in the IBSR dataset

	3D-CNN	FastSurfer	FSL-FIRST
Dice coefficient relative to manual expert delineation, mean (std)	0.86 (0.02)	0.86 (0.02)	0.87 (0.02)
Hausdorff distance in mm, mean (std)	4.51 (0.91)	5.18 (1.03)	4.55 (0.90)
THALV - IBSR THALV [mL], mean (std)	0.61 (0.76)	0.35 (1.19)	1.26 (0.77)
Pearson correlation coefficient THALV versus IBSR THALV (*p* value)	−0.44 (*p* < 0.0005)	0.56 (*p* < 0.0005)	−0.28 (*p* < 0.0005)

### Intra- and inter-scanner variability

**Fig. 5:**
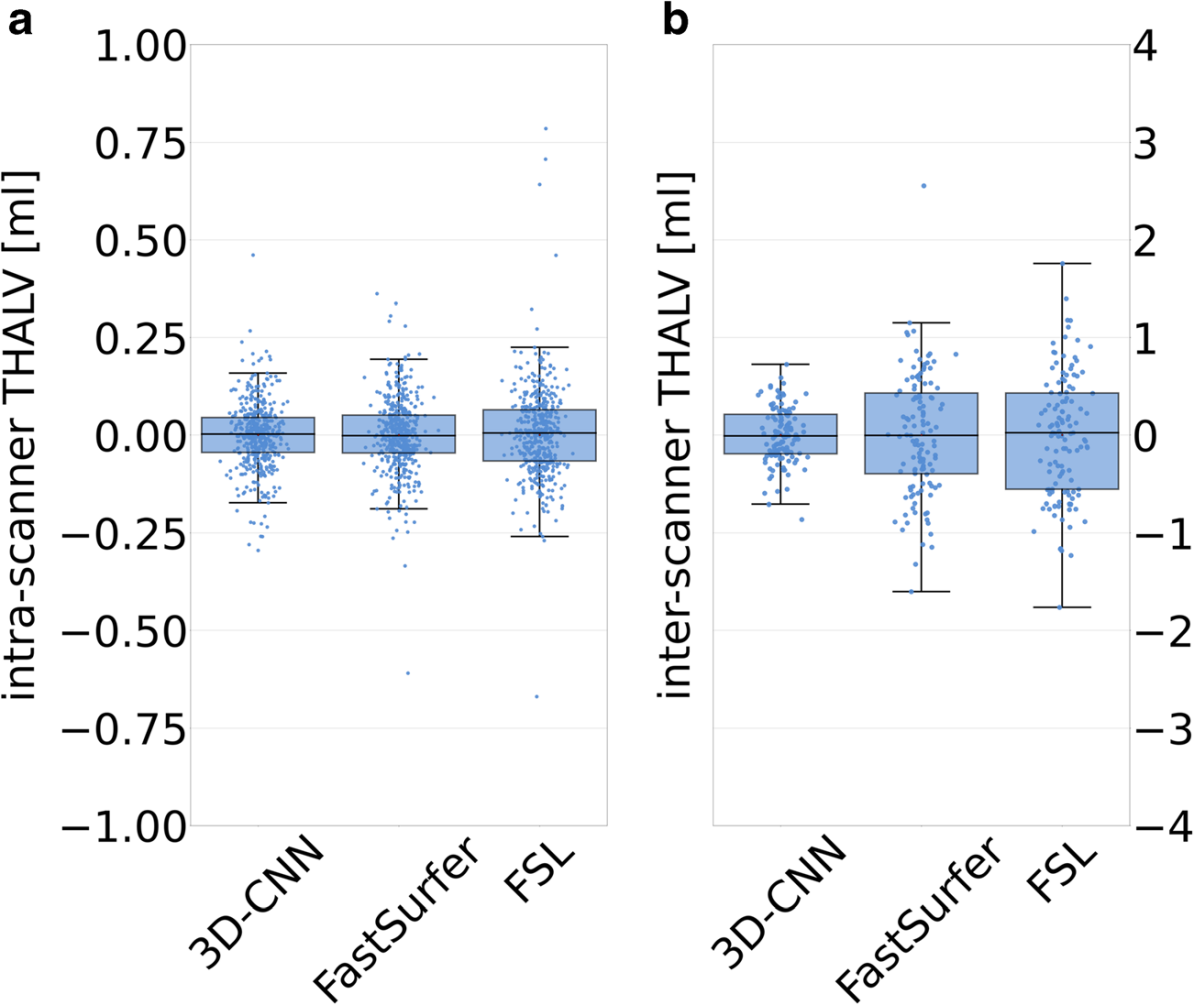
Intra- (**a**) and inter-scanner (**b**) variability of THALV estimated with 3D-CNN, FastSurfer, and FSL in the FTHP dataset

Results on inter- and intra-scanner variability are summarized in Table 3 and Fig. 5. The variance of the intra-scanner residuals of THALV in the FTHP dataset was significantly smaller for 3D-CNN (std = 0.08 mL, *p* < 0.0005) and FastSurfer (std = 0.09 mL, *p* = 0.012) compared to that in FSL (std = 0.15 mL). The reduction of the intra-scanner residuals with 3D-CNN compared to FastSurfer missed the Bonferroni-corrected significance threshold (*p* = 0.023 > 0.017). The variance of the inter-scanner residuals of THALV was significantly smaller for 3D-CNN (std = 0.28 mL) compared to that for FastSurfer (std = 0.62 mL, *p* < 0.0005) and FSL (std = 0.63 mL, *p* < 0.0005). The difference of the variance of the inter-scanner residuals between FastSurfer and FSL was not significant (*p* = 0.816).

**Table 3:** Intra- and inter-scanner variability of thalamus volume estimates in the FTHP dataset

	Intra-scanner			Inter-scanner		
	3D-CNN	FastSurfer	FSL	3D-CNN	FastSurfer	FSL
std of THALV residuals [mL]	0.08	0.09	0.15	0.28	0.62	0.63
50th percentile of absolute value of THALV residuals [mL]	0.04	0.05	0.07	0.20	0.41	0.48
95th percentile of absolute value of THALV residuals [mL]	0.16	0.18	0.20	0.57	1.11	1.18

### Single subject analysis

**Fig. 6:**
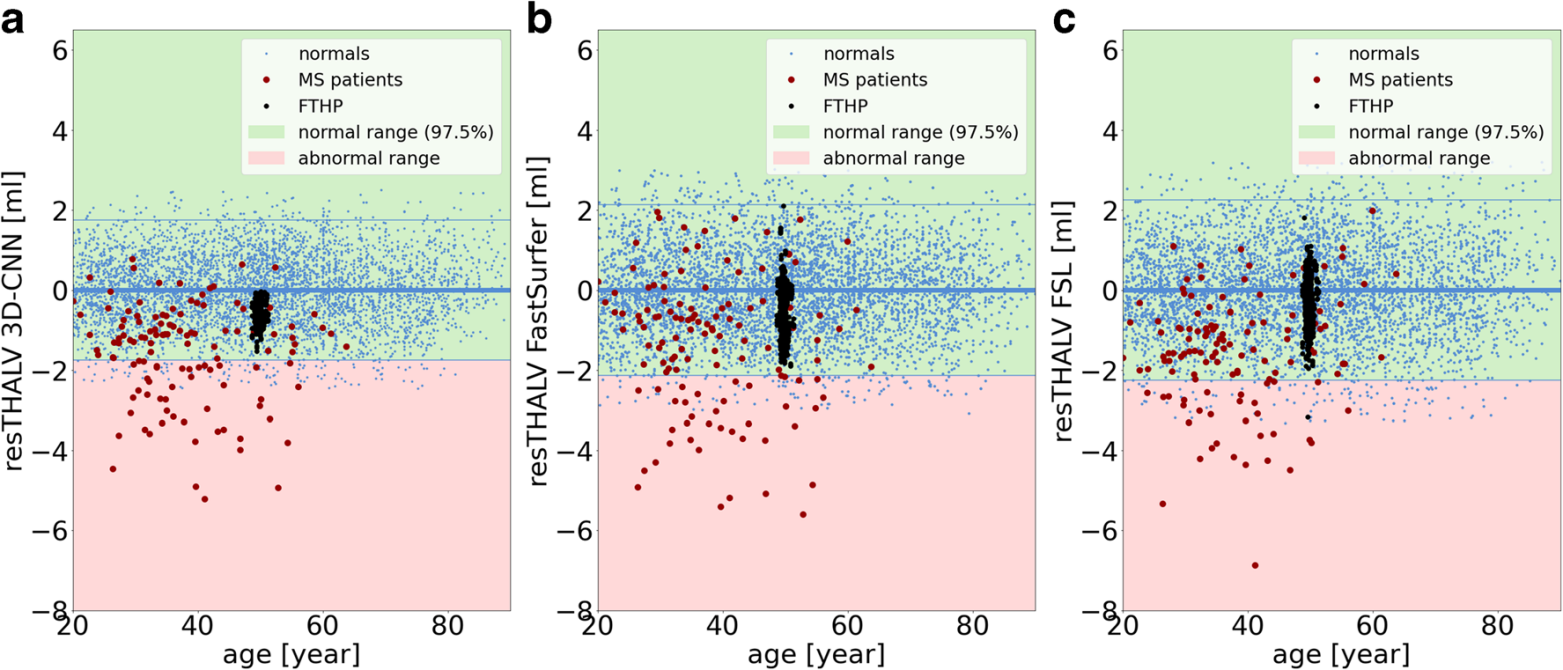
The three plots show the resTHALV of the 4872 subjects in the normal database (blue dots) for 3D-CNN (**a**), FastSurfer (**b**), and FSL (**c**). The red dots indicate the MS patients. The black dots represent resTHALV for the frequently traveling human phantom (FTHP) dataset

Scatter plots of TIV- and age-corrected resTHALV versus age in the normal database are shown in Fig. 6. The standard deviation of resTHALV in the normal database was 0.88 mL, 1.09 mL, and 1.15 mL for 3D-CNN, FastSurfer, and FSL (Table 4).

**Table 4:** Between-subjects variability of thalamus volume estimates in the normal database and proportion of MS patients with significantly reduced thalamus volume

	3D-CNN	FastSurfer	FSL
std of resTHALV in the normal database [mL]	0.88	1.09	1.15
Proportion of MS patients with reduced THALV [%]	23.8	15.9	11.1

The proportion of MS patients with reduced THALV was 24%, 16%, and 11% for 3D-CNN, FastSurfer, and FSL (Table 4; Fig. 6).

## Discussion

Thalamus segmentation with the 3D-CNN was similarly accurate as with FastSurfer and FSL, but it was considerably more robust against repeat scanning, particularly against repeat scanning on different MRI scanners: inter-scanner variability of the estimated thalamus volume was lower by more than a factor two with the 3D-CNN (0.28 mL; Table 3) than with FastSurfer (0.62 mL) and FSL (0.63 mL). This might be explained by the large size and the heterogeneity of the training dataset comprising 1975 T1w-MRI of 1975 different patients from 170 different MRI scanners. For comparison, FastSurfer was trained on 160 T1w-MRI [16]. Furthermore, massive data augmentation was used for 3D-CNN training including adding random bias fields and noise in addition to standard augmentation. This might have forced the 3D-CNN to focus on the relevant features rather than tuning results too strongly to specific image characteristics of a given scanner or acquisition sequence.

Reduction of intra- and inter-scanner variability might be achieved not only by reduction of variance of no interest (associated with scanner-related variability of image characteristics) but also by reduction of variance of interest associated with actual between-subjects variability (a method that simply returns a thalamus volume of 15 mL for all subjects performs perfectly well with respect to intra- and inter-scanner variability, but clearly is useless). The higher proportion of patients with significantly reduced thalamus volume in the MS dataset when using the 3D-CNN (24%) compared to FastSurfer (16%) and FSL (11%) suggests that this is not the case with the novel 3D-CNN. It rather suggests that the improved intra- and inter-scanner stability of the 3D-CNN resulted in increased sensitivity for the detection of thalamus atrophy in MS [31, 32]. This is further supported by the fact that the disease severity in the MS patients was significantly correlated with the THALV estimates from the 3D-CNN and from FastSurfer but not with those from FSL (section “Impact of the segmentation method on the correlation between the thalamus volume and disease severity in MS” in the supplementary material). However, the correlations were weak (Pearson correlation coefficient ≤ 0.29) and therefore should be interpreted cautiously. The low strength of the observed correlations is in line with previous studies that reported either a significant weak correlation between EDSS and thalamus volume (e.g., Datta et al: *r* = −0.133, *p* < 0.001, 924 MS patients [31]) or lack of significant correlation (e.g., Tommasin et al: Spearman correlation coefficient = 0.07, *p* ≥ 0.01, 163 MS patients [33]).

Intra- and inter-scanner variability of MRI-based regional brain volume estimates are often reported as absolute (in mL) or relative differences (in %) [12, 13]. In the context of clinical applications of MRI-based brain volumetry, in which individual volume estimates are compared to a normal database, it might be more appropriate to specify intra- and inter-scanner variability in relation to the between-subjects variability in the normal database. Figure 6 demonstrates that the inter-scanner variability of the thalamus volume estimates in the FTHP dataset covers a significant fraction of the 95% CI of between-subjects variability in the normal database. This suggests that inter-scanner variability of thalamus volume estimates significantly contributes to the between-subjects variability in a normal database comprising scans from different scanners. Thus, reduction of inter-scanner variability results in relevant reduction of normal between-subjects variability. This is highly relevant, because it is expected to result in greater power for the detection of disease-related alterations. The ratio of inter-scanner variability (in the FTHP dataset) to between-subjects variability in the normal database was 0.28/0.88 = 0.32 for 3D-CNN, 0.62/1.09 = 0.57 for FastSurfer, and 0.63/1.15 = 0.55 for FSL (Tables 3 and 4). The larger fraction of MS patients with reduced thalamus volume according to the 3D-CNN estimates is in line with the lower inter-scanner-to-between-subjects variability ratio for the 3D-CNN.

In clinical applications of MRI-based brain volumetry, reliable detection of disease-related alterations is more important than estimation of the actual volume of the brain region of interest with highest possible accuracy (but lower precision). However, the 3D-CNN provides also high accuracy for thalamus segmentation as indicated by the mean Dice coefficient of 0.86 relative to manual expert delineation. Sitter and co-workers reported Dice coefficients between repeat manual thalamus segmentation by the same expert ranging from 0.87 to 0.91 [34].

The 3D-CNN resulted in some overestimation of the volume of small thalami compared to manual segmentation (Fig. 3A), similar to other automatic methods [10]. The reason is unclear. This requires further investigation, because it might limit the detection of mild thalamus atrophy in early disease stages by automatic thalamus volumetry.

Thalamus masks automatically generated by FSL were used as ground truth in the present study. The use of conventional automatic methods to generate ground truth for deep learning has been explored previously [35]. Other freely available software packages also provide segmentation of the thalamus (e.g., FreeSurfer [25], CAT12 [36], volBrain [37]) and, therefore, also could have been used for generation of the ground truth. The rationale for selecting FSL was that it provided the best agreement with manual expert delineation of the thalamus in a study by Sitter and co-workers [34]. Manual thalamus delineation by experts would have been preferred for the network training but was not feasible given the large size of the training set (*n* = 1975).

Considering the architecture of the 3D-CNN, a U-net structure and the full 3-dimensional T1w-MRI as input were chosen. U-net-based CNNs are the current state-of-the-art for segmentation tasks [38], also compared to other CNN architectures [39]. FastSurfer [16], a recent development based on QuickNAT [40], deploys a 2D-U-net that outperformed a 3D-U-net tested by the developers of FastSurfer. A possible explanation provided by the authors is that their 3D-U-net used small volume patches due to GPU memory constraints [16]. In the present study, the 3D-CNN used rather large patches covering approximately 2/3 of the brain (Fig. 1). Therefore, sufficient context information was contained in each patch. Furthermore, instance normalization was used in the present study instead of the standard batch normalization to cope with the limited batch size. We hypothesize that the 3D design allowed the 3D-CNN to become particularly robust with respect to camera-specific variability of image characteristics.

No manual correction of the automatic segmentation results was performed in this study. Clinical use of automatic thalamus volumetry should include visual quality control of the segmentation and manual correction if required.

In conclusion, the proposed 3D-CNN provides accurate thalamus segmentation that is particularly robust with respect to MRI scanner and protocol changes. This might improve the sensitivity to detect disease-related thalamus atrophy in multi-site/multi-scanner settings without the need for scanner-specific normal databases, despite the fact that the 3D-CNN tended to slightly overestimate the volume of small thalami. Further improvement might be achieved by training with manual thalamus segmentation as ground truth. The 3D-CNN provides segmentation of the left and the right thalamus. Thalamus parcellation into nuclei and subnuclei would require further specific training.

## Supplementary information

ESM 1 (DOCX 483 kb)

## Abbreviations

2D/3D: Two/three-dimensional
95% CI: 95% confidence interval
CNN: Convolutional neural network
FastSurfer: 2D-CNN software library
FSL: Software library https://fsl.fmrib.ox.ac.uk/fsl/fslwiki/FIRST
FTHP: Frequently traveling human phantom
IBSR: Internet Brain Segmentation Repository
MRI: Magnetic resonance imaging
MS: Multiples sclerosis
std: Standard deviation
THALV: Thalamus volume
TIV: Total intracranial volume

## Declarations

### Guarantor

The scientific guarantor of this publication is Roland Opfer.

### Statistics and biometry

One of the authors (RB) has significant statistical expertise.

### Methodology

• retrospective

• cross-sectional study

• performed at one institution

## References

[1] Raji A, Ostwaldt AC, Opfer R, Suppa P, Spies L, Winkler G (2018). MRI-based brain volumetry at a single time point complements clinical evaluation of patients with multiple sclerosis in an outpatient setting. Front Neurol.

[2] Zivadinov R, Havrdová E, Bergsland N (2013). Thalamic atrophy is associated with development of clinically definite multiple sclerosis. Radiology.

[3] Amin M, Ontaneda D (2020). Thalamic injury and cognition in multiple sclerosis. Front Neurol.

[4] Filippi M, Rocca MA, Pagani E (2014). Placebo-controlled trial of oral laquinimod in multiple sclerosis: MRI evidence of an effect on brain tissue damage. J Neurol Neurosurg Psychiatry.

[5] Schoonheim MM, Popescu V, Rueda Lopes FC (2012). Subcortical atrophy and cognition: sex effects in multiple sclerosis. Neurology.

[6] Glaister J, Carass A, NessAiver T (2017). Thalamus segmentation using multi-modal feature classification: validation and pilot study of an age-matched cohort. Neuroimage.

[7] Iglesias JE, Insausti R, Lerma-Usabiaga G (2018). A probabilistic atlas of the human thalamic nuclei combining ex vivo MRI and histology. Neuroimage.

[8] Iglesias JE, Van Leemput K, Golland P, Yendiki A (2019). Joint inference on structural and diffusion MRI for sequence-adaptive Bayesian segmentation of thalamic nuclei with probabilistic atlases. Inf Process Med Imaging.

[9] Su JH, Thomas FT, Kasoff WS (2019). Thalamus Optimized Multi Atlas Segmentation (THOMAS): fast, fully automated segmentation of thalamic nuclei from structural MRI. Neuroimage.

[10] Burggraaff J, Liu Y, Prieto JC (2021). Manual and automated tissue segmentation confirm the impact of thalamus atrophy on cognition in multiple sclerosis: a multicenter study. Neuroimage Clin.

[11] Kruggel F, Turner J, Muftuler LT, Alzheimer’s Disease Neuroimaging I (2010). Impact of scanner hardware and imaging protocol on image quality and compartment volume precision in the ADNI cohort. Neuroimage.

[12] Cover KS, van Schijndel RA, van Dijk BW (2011). Assessing the reproducibility of the SienaX and Siena brain atrophy measures using the ADNI back-to-back MP-RAGE MRI scans. Psychiatry Res.

[13] Opfer R, Suppa P, Kepp T, Spies L, Schippling S, Huppertz HJ (2016). Atlas based brain volumetry: how to distinguish regional volume changes due to biological or physiological effects from inherent noise of the methodology. Magn Reson Imaging.

[14] Bernal J, Kushibar K, Asfaw DS (2019). Deep convolutional neural networks for brain image analysis on magnetic resonance imaging: a review. Artif Intell Med.

[15] Krüger J, Opfer R, Gessert N (2020). Fully automated longitudinal segmentation of new or enlarged multiple sclerosis lesions using 3D convolutional neural networks. Neuroimage Clin.

[16] Henschel L, Conjeti S, Estrada S, Diers K, Fischl B, Reuter M (2020). FastSurfer - a fast and accurate deep learning based neuroimaging pipeline. Neuroimage.

[17] Wenzel M, Milletari F, Kruger J (2019). Automatic classification of dopamine transporter SPECT: deep convolutional neural networks can be trained to be robust with respect to variable image characteristics. Eur J Nucl Med Mol Imaging.

[18] Patenaude B, Smith SM, Kennedy DN, Jenkinson M (2011). A Bayesian model of shape and appearance for subcortical brain segmentation. Neuroimage.

[19] Ronneberger O, Fischer P, Brox T (2015) U-net: Convolutional networks for biomedical image segmentation. International conference on medical image computing and computer-assisted intervention. Springer, pp 234-241

[20] Çiçek Ö, Abdulkadir A, Lienkamp SS, Brox T, Ronneberger O (2016) 3D U-Net: learning dense volumetric segmentation from sparse annotation. International conference on medical image computing and computer-assisted intervention. Springer, pp 424-432

[21] Krüger J, Ostwaldt AC, Spies L et al (2021) Infratentorial lesions in multiple sclerosis patients: intra- and inter-rater variability in comparison to a fully automated segmentation using 3D convolutional neural networks. Eur Radiol. 10.1007/s00330-021-08329-3 10.1007/s00330-021-08329-3 34643779

[22] Coronado I, Gabr RE, Narayana PA (2020) Deep learning segmentation of gadolinium-enhancing lesions in multiple sclerosis. Mult Scler. 10.1177/1352458520921364 10.1177/1352458520921364 PMC7680286 32442043

[23] Malone IB, Leung KK, Clegg S (2015). Accurate automatic estimation of total intracranial volume: a nuisance variable with less nuisance. Neuroimage.

[24] Ashburner J, Friston KJ (2005). Unified segmentation. Neuroimage.

[25] Fischl B, Salat DH, Busa E (2002). Whole brain segmentation: automated labeling of neuroanatomical structures in the human brain. Neuron.

[26] Pell GS, Briellmann RS, Chan CH, Pardoe H, Abbott DF, Jackson GD (2008). Selection of the control group for VBM analysis: influence of covariates, matching and sample size. Neuroimage.

[27] Schippling S, Ostwaldt A-C, Suppa P et al (2017) Global and regional annual brain volume loss rates in physiological aging. J Neurol. 10.1007/s00415-016-8374-y:1-9 10.1007/s00415-016-8374-y 28054131

[28] Opfer R, Krüger J, Spies L et al. (2022) Single-subject analysis of regional brain volumetric measures can be strongly influenced by the method for head size adjustment. Neuroradiology 64(10):2001–2009 10.1007/s00234-022-02961-6 PMC9474386 35462574

[29] Muhlau M, Wohlschlager AM, Gaser C (2009). Voxel-based morphometry in individual patients: a pilot study in early Huntington disease. AJNR Am J Neuroradiol.

[30] Opfer R, Ostwaldt AC, Walker-Egger C et al (2018) Within-patient fluctuation of brain volume estimates from short-term repeated MRI measurements using SIENA/FSL. J Neurol. 10.1007/s00415-018-8825-8 10.1007/s00415-018-8825-8 29549466

[31] Datta S, Staewen TD, Cofield SS (2015). Regional gray matter atrophy in relapsing remitting multiple sclerosis: baseline analysis of multi-center data. Mult Scler Relat Disord.

[32] Calabrese M, Reynolds R, Magliozzi R (2015). Regional distribution and evolution of gray matter damage in different populations of multiple sclerosis patients. PLoS One.

[33] Tommasin S, Cocozza S, Taloni A (2021). Machine learning classifier to identify clinical and radiological features relevant to disability progression in multiple sclerosis. J Neurol.

[34] de Sitter A, Verhoeven T, Burggraaff J (2020). Reduced accuracy of MRI deep grey matter segmentation in multiple sclerosis: an evaluation of four automated methods against manual reference segmentations in a multi-center cohort. J Neurol.

[35] Huo Y, Plassard AJ, Carass A (2016). Consistent cortical reconstruction and multi-atlas brain segmentation. Neuroimage.

[36] Mutsaerts H, Petr J, Groot P (2020). ExploreASL: an image processing pipeline for multi-center ASL perfusion MRI studies. Neuroimage.

[37] Carass A, Cuzzocreo JL, Han S (2018). Comparing fully automated state-of-the-art cerebellum parcellation from magnetic resonance images. Neuroimage.

[38] Dou Q, Yu L, Chen H (2017). 3D deeply supervised network for automated segmentation of volumetric medical images. Medical image analysis.

[39] Dolz J, Desrosiers C, Ben Ayed I (2018). 3D fully convolutional networks for subcortical segmentation in MRI: a large-scale study. Neuroimage.

[40] Guha Roy A, Conjeti S, Navab N, Wachinger C (2019). QuickNAT: a fully convolutional network for quick and accurate segmentation of neuroanatomy. Neuroimage.

