# Supplementary material for: Automatic segmentation of the thalamus using a massively trained 3D convolutional neural network: higher sensitivity for the detection of reduced thalamus volume by improved inter-scanner stability
Source: Eur Radiol. 2022 Oct 20;33(3):1852–61. doi: 10.1007/s00330-022-09170-y (PMC9935653; doi:10.1007/s00330-022-09170-y)
Supplement: Supplementary file 1 — (DOCX 483 kb) [file 330_2022_9170_MOESM1_ESM.docx]

**Supplementary Information**

Title: Automatic segmentation of the thalamus using a massively trained 3D convolutional neural network: higher sensitivity for the detection of reduced thalamus volume by improved inter-scanner stability

**Three-dimensional convolutional neural network (3D-CNN)**

The 3D-CNN was implemented in python 3.7 using the deep learning framework pytorch 1.3. Training and validation were performed on a Linux Debian 10 machine with a 2.4GHz CPU (Intel Xeon Silver 10-Core) with a GPU Quadro P5000 with 16GB of memory.

The architecture of the 3D-CNN is shown in Figure 1 in the manuscript. The CNN follows a fully convolutional encoder-decoder (U-net-like) architecture with 3D convolutions with 3x3x3 kernel size. The encoder reduces the spatial feature map size four times (using convolution with stride 2) and doubles the feature map number with each reduction. Starting with 16 feature maps of size 128x128x128 in the first layer, this leads to 256 maps of size 8x8x8 in the last encoder layer. The decoder uses convolution layers, followed by nearest-neighbour up-sampling and deep supervision in three layers [1]. A feature concatenation was employed for the long-range connections between encoder and decoder. A leaky ReLU [2] was used as activation function in each layer. Due to the rather large patches of 160x160x160 voxels covering about 2/3 of the brain, batch size of one was selected. Therefore, instance normalization, a special case of group normalization, was used instead of batch normalization.

**Pre-processing of the volume images**

The only pre-processing of the volume images was resampling into isotropic 1mm x 1mm x 1mm voxels. Bias field correction, intensity normalization, spatial alignment and other pre-processing steps that attempt to make the data more homogenous were not performed. The rationale for this was that training the network with a particularly large and heterogeneous dataset might improve its robustness with respect to camera-specific variability of image characteristics.

**Data augmentation**

A random combination of the following augmentation techniques was used during the training to increase the heterogeneity of the training dataset:

- left-right flipping
- rotation around the x-, y- or z-axis by an angle randomly chosen between -10 and 10 degrees
- translation in x-, y- or z-direction by a distance randomly chosen between -5 and 5 mm
- voxelwise adding of Gaussian random noise with zero mean and variance randomly chosen between 0 and 0.0001 (voxel intensities were normalized to range between 0 and 1)
- adding a bias field obtained as follows. A transaxial reference plane was randomly selected from the 3D volume image. For the remaining transaxial planes the signed distance d in mm was computed. All voxel intensities in a given plane were multiplied by (1 + r*d/max(abs(d))) were d is the signed distance of the considered plane from the reference plane, max(abs(d)) is the maximum absolute distance of all planes, and r is a random number between 0 and 1.

**Training and application of the 3D-CNN**

The 3D-CNN was trained from scratch, that is, no pre-trained weights were used. The Adam optimizer (“Adaptive Moment Estimation”) was used, a version of stochastic gradient descent [3], with a Dice loss function (sum of Dice over all classes) for Ne = 100 epochs. A starting learning rate of α_0_ = 10^-4^ with decay according to α = α_0_ (1 – epoch/Ne ) 0.9 was used.

For the application of the 3D-CNN to a given T1w volume image, eight evenly distributed overlapping crops of 160x160x160 voxels were taken from the volume image. For each crop, the predicted class values were computed and merged to the entire volume by taking the mean class values in the overlapping regions.

The automatically generated output of the 3D-CNN contains probability values between 0 and 1 for each class. A discrete segmentation mask was generated by assigning the class label of the class with the highest probability at each voxel.

The training of the 3D-CNN was carried out in a two-step approach. In the first step, all 1,975 volume images of the training dataset were used for the training. The resulting preliminary 3D-CNN was used for thalamus segmentation in the same training dataset. The 3D-CNN thalamus masks were compared with the FSL ground truth masks using the Dice coefficient. The Dice coefficient was < 0.5 in 73 of the 1,975 volume images (3.7%). Visual inspection revealed complete segmentation failure by FSL in each of these cases, most likely due to failure of the initial registration to the atlas space by FSL. This is a known limitation of FSL, Cavedo et al. reported a failure rate of 1.8% [4]. The 73 volume images with FSL segmentation failure did not provide useful ground truth and therefore were excluded from the second training step, in which the final 3D-CNN was trained from scratch with the remaining 1,902 training cases.

**Impact of the segmentation method on the correlation between the thalamus volume and disease severity in MS**

The figure below shows scatter plots of the TIV- and age-corrected residual of the thalamus volume (resTHALV) versus the Expanded Disability Status Scale (EDSS), separately for the three segmentation methods. The dotted lines represent the result of linear regression. Pearson correlation analysis revealed r=-0.20 (p=0.02), r=-0.29 (p=0.001), and r=0.03 (p=0.67) for resTHALV from 3D-CNN, FastSurfer and FSL, respectively. Thus, disease severity in the MS sample was (inversely) correlated with the THALV estimates from the 3D-CNN and from FastSurfer, but not with those from FSL.


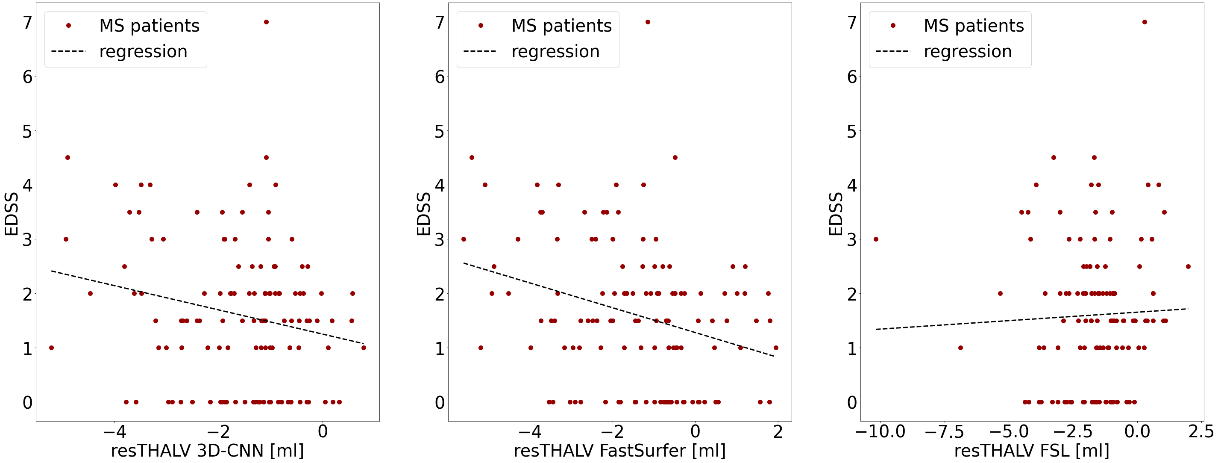


**References**

1 Dou Q, Yu L, Chen H et al (2017) 3D deeply supervised network for automated segmentation of volumetric medical images. Medical image analysis 41:40-54

2 Maas AL, Hannun AY, Ng AY (2013) Rectifier nonlinearities improve neural network acoustic modelsProc icml. Citeseer, pp 3

3 Kingma DP, Ba J (2014) Adam: A method for stochastic optimization. arXiv preprint arXiv:14126980

4 Cavedo E, Suppa P, Lange C et al (2017) Fully Automatic MRI-Based Hippocampus Volumetry Using FSL-FIRST: Intra-Scanner Test-Retest Stability, Inter-Field Strength Variability, and Performance as Enrichment Biomarker for Clinical Trials Using Prodromal Target Populations at Risk for Alzheimer's Disease. J Alzheimers Dis 60:151-164


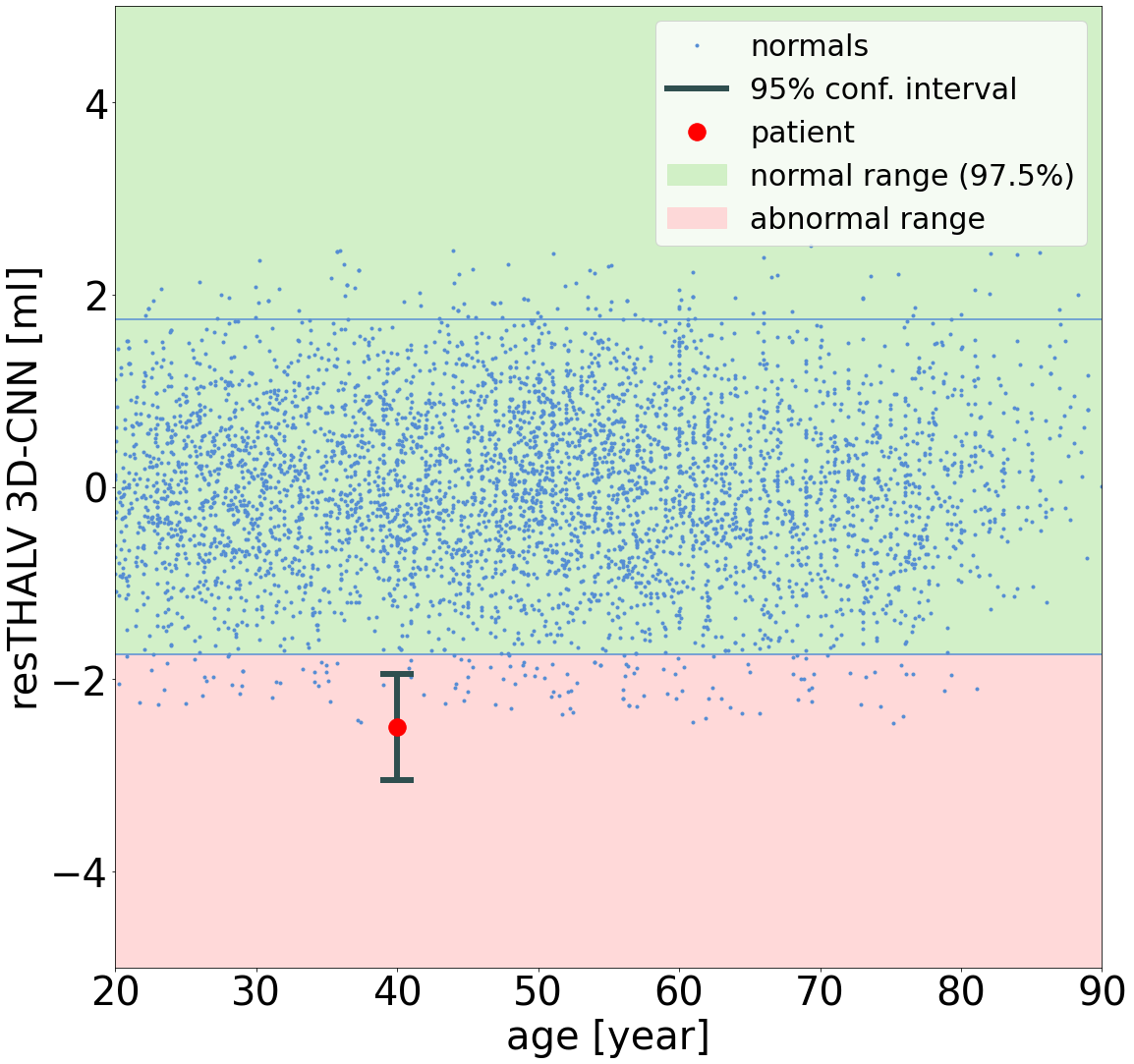


**Supplementary Figure 1.** Illustration of how resTHALV (residual thalamus volume) measurements were interpreted for individual patients. The patient’s THALV was consídered reduced if the 95%-CI of the patient’s resTHALV was below the 95%-CI of resTHALV in the normal database.
